# Thinking of me: Self-focus reduces sharing and helping in seven- to eight-year-olds

**DOI:** 10.1371/journal.pone.0189752

**Published:** 2018-01-10

**Authors:** Sandra Weltzien, Lauren E. Marsh, Bruce Hood

**Affiliations:** School of Experimental Psychology, University of Bristol, Bristol, United Kingdom; Swinburne University of Technology, AUSTRALIA

## Abstract

By 7-to 8-years of age, most children readily adhere to prosocial norms aimed at benefiting others through giving up time and effort (helping) or resources (sharing). Two studies explored whether sharing and helping by 7-to 8-year olds (N = 180) could be influenced by priming children’s attention on themselves or their friends through a semi-structured interview. Results revealed that self-priming led to reductions in both sharing and helping compared to friendship-priming or a control condition. These findings are considered as indicative of the fragile state of prosocial behaviours at this age that can be easily shifted towards more selfish biases by simple priming.

## Introduction

Most human beings are willing to give up resources, time, and effort to benefit others across a range of interactions. In traditional models of moral development (e.g., [1], [2], [3], [4]) there is general agreement that with age, children's reasoning develops from being more hedonistic and self-concerned, to becoming more other-oriented and concerned with adherence to social norms and acceptance from others. There is also abundant empirical evidence that prosocial behaviours increase with age [5], [6], [7], [8], [9]. However, not all prosocial behaviours show the same developmental pattern. For example, sharing and helping are both prosocial behaviours that exhibit different developmental trajectories. While there may be procedural issues when comparing helping and sharing, naturalistic observational studies (e.g. [10], [11], [12]) indicate that the differences reflect true dispositions in most children.

Helping is an early emerging prosocial behaviour [13], [14]. Soon after their first birthday, infants will pick up and return an object accidentally dropped on the floor, and open cabinets for people if their hands are full [13], [15]. They do this irrespective of praise or encouragement, and even if the helping is costly, for example requiring the child to leave a fun activity or surmount obstacles in order to help [15]. To deliver the appropriate help in these situations, infants must understand the behaviour of others as goal-oriented, and be motivated to help them achieve their aim. Such findings have fuelled the argument that humans begin life as indiscriminate helpers [16], and thus that children may have a “natural tendency to help other persons” [13].

In contrast, spontaneous sharing behaviour seems to emerge later in development. While there are some examples of simple resource distribution during infancy [17], [18], [19], [20], [21], these are typically limited to direct requests (but see [22]). There is also evidence that infants may hold certain expectations regarding how resources are typically distributed. For example, 15-month-old infants demonstrate sensitivity to fairness violations [23], and by two years of age, children expect a fair split of resources between individuals when the distributer is a third party, but expect an uneven split if unequal effort has been contributed [24]. Yet, despite these early expectations of fairness, young children typically behave selfishly in situations that require relinquishing their own possessions, resulting in a disparity between their own sharing behaviour and the principles of sharing they endorse for others (e.g., [25], [26], [27].

Smith and colleagues [27] investigated the gap between sharing standards and sharing behaviour in 3- to 8-year-olds. At all ages, children endorsed norms for sharing, but 3- to 6-year-olds still favoured themselves in real sharing situations. When explaining their sharing behaviour, older children focused spontaneously and explicitly on sharing norms, whereas the younger children focused on themselves and their own desires [27]. This could suggest that the development of sharing norms must overcome an initial self-interested bias to optimise own gain. In other words, young children are aware of sharing norms, but these principles appear overridden by self-focus before age 7. The attenuation of selfish behaviour with age is typically explained as a consequence of socialization. As children move from an informal preschool setting to formal schools, they may gradually learn and internalize the normative rules endorsed by authority figures such as teachers and other adults (e.g., [25], [7], see [28] for cross-cultural evidence). This is also a period where children become increasingly sensitive to others opinions about themselves, which may increase their desire and motivation to appear generous and behave in a normatively appropriate way [29], [30].

In summary, children appear to show an early appreciation of both sharing and helping norms (e.g. [23], [24]) but whereas helping is often enacted at an early age, sharing is typically not [31], [32]. Why is this? The disparity between sharing and helping may be due to a number of differences between the two behaviours. Sharing is arguably more costly, as it usually requires the reallocation and loss of resources that are already in one’s possession. However, even in situations where there is no personal cost, young children are still reluctant to share unless specifically requested to do so [19]. Helping is not so much a loss of physical resources, but rather of time and effort not yet expended. While both sharing and helping benefit others, it could be that the cost of relinquishing personal resources weighs more heavily than the cost of assistance. It could also be that helping is more other-directed in that it requires interpreting another’s immediate need for assistance. Moreover, the likelihood of helping is influenced by prior positive social engagement with the individual who subsequently requires assistance. Infants are more likely to help another person if that person has previously engaged in mutual play [33] or danced in synchrony [34]. These findings are consistent with the interpretation that early helping is a social-interactional behaviour that “develops through reciprocal interactions” which by definition requires coordinating with another [35].

One way to explore the extent to which sharing and helping are driven by self- and other-focus motivations is to examine whether these behaviours can be enhanced or diminished through external manipulations, such as priming. Priming refers to the activation of a schema or category that can have an impact on subsequent reasoning and behaviour. Hebb [36] proposed that such internal mental representations remain latent, but can be elicited by both internal and external triggers. For example, adult studies have shown that priming the concept of helping increases willingness to assist in a subsequent task [37]. Mental representations also include complex aspects of the self-concept, and recent studies with adults have demonstrated that the salience of specific self-construals can be manipulated through priming [38], [39], [40], [41]. Importantly, it has been found that activation of independent and interdependent self-construals can lead to transformation of motives, which in turn can affect levels of prosocial behaviours. For example, adults primed with independence words such as "individual" were more selfish in subsequent sharing tasks compared to adults primed with interdependence words such as "friendship" [41].

Social development research, however, has rarely used priming despite evidence that this technique is promising for helping researchers untangle causal connections between children’s mental representations and children’s social development [42]. Moreover, the existing research on priming prosociality in children has typically focused on the relationship between empathic concern and prosocial behaviours (e.g., [43], [44], [45]). Here, we adopted a self-construal priming paradigm developed by Hood, Weltzien, Marsh and Kanngiesser [46] which revealed that children changed their evaluation of personal possessions when primed to think about themselves. Specifically, following self-focus, it was found that participants increased their evaluation of an owned object, and were less willing to trade that object upon request from an experimenter compared to participants who completed a neutral control task. Conversely, when participants had completed a friend-focus task, they were more willing to subsequently trade with the experimenter. As these were identical objects, there was no advantage in trading other than complying with the request. If willingness to trade in this context can be considered a pro-social behaviour, we predicted that similar self-construal priming would also influence other pro-social behaviours.

Despite different developmental trajectories, children will both share and help reliably by 7 years of age. But how substantiated are these behaviours? In the current line of work, we investigated whether self- and friendship-priming would differentially influence these two prosocial behaviours. More specifically, in Study 1 we examined the effects of self- and friendship-priming on sharing behaviour using a Dictator Game [47]. We adapted the self-construal priming paradigm previously used with 3- to 4-year-olds [46] into one more suitable for primary school children. This was done using a semi-structured interview and drawing task that either focused children’s attention on themselves, their relationship with a close friend, or on a neutral farm (control). Study 2 explored the effects of the same priming manipulations on a helping task.

It was predicted that if sharing requires children to overcome a self-interest bias, then children primed towards self-focus should be more reluctant to share their resources compared to the control-condition. We also sought to evaluate whether sharing could be enhanced through friendship-priming, although due to the self-focused nature of sharing we did not hold strong predictions regarding the effectiveness of this priming manipulation. Helping, on the other hand, is enacted early in development across a range of situations, suggesting that there may not be such a strong bias for self-interest related to this behaviour. If this holds true, then helping should be relatively resilient to self-priming. Moreover, it has been found that young children primed with stimuli that evoke affiliation are more inclined to help a person in need than children primed towards individuality [48]. Thus, in the present study we predicted that focusing children on their relationship with others should increase their helping behaviour compared to the control by making them more other-oriented.

## Study 1: Sharing

The study protocol was approved by the Faculty of Science Research Ethics Committee at the University of Bristol. Informed consent was obtained in written form from the parents of all children who participated in this study. All children provided verbal agreement that they wished to partake in the research.

## Method

### Participants

Ninety 7-to 8-year olds (*M*_*age*_ *=* 94.17 months, *SD*_*age*_ *=* 7.47, range = 84–107 months; 40 males) participated in Study 1. This sample size was selected based on a power calculation conducted on the effect size taken from Guinote, Cotzia, Sandhu and Siwa [49] which reports an effect of social status on generosity in a Dictator Game in 5-year olds. That study used a social manipulation of pairings of children playing the Dictator Game, which is the most similar design to the present sharing study that we could find. Based on the calculated effect size of f = .4, a sample of 27 children per group should yield 95% power. Thus, with 30 children in three groups, we have 97.5% power to detect an effect of the same size in the current study. Four additional children were tested but excluded from analysis because they failed to pass one or more of the control questions. All children were tested individually in quiet rooms at the Bristol Cognitive Development Centre or at the At-Bristol science centre. A majority of the children were of Western descent (92%) and came from middle-class, educated families.

### Procedure

#### Sticker allocation

Each testing session began by the child choosing six of ten colourful animal stickers to keep. Once the selection was made, the remaining stickers were removed from sight. The child’s selected stickers remained in view, but out of reach, on the table.

#### Priming-procedure

Each child took part in one of three priming conditions: self-priming, friendship-priming, or a neutral control condition. Using a semi-structured script, the experimenter asked a series of questions about the child (self-priming), a best friend and their relationship (friendship-priming), or a farm (neutral-priming), whilst drawing a picture based on the child’s description. The self- and friendship-priming interviews had 20 similar questions related to appearances, preferences, clothing and activities. During self-priming, the experimenter used second person singular pronouns (you, your, yours, child’s name) whenever apt in order to prime notions of independence and steer the child’s attention towards his or her self. In the friendship-priming condition, the experimenter used third person pronouns (he, she, him, her, friend’s name) and second person plural pronouns (you, your) whenever apt to prime notions of interdependence and steer the child’s attention towards the friend and their relationship. In the neutral-priming condition, the experimenter was careful to avoid use of personal pronouns (for full priming scripts and experimental instructions, see supporting information (S1 File).

#### Dictator game

In the present experiment, two envelopes were introduced to the child; one brown and one white. The child was informed that the brown envelope belonged to him or her, and that everything put into this envelope could be taken home. The child’s name was written on the envelope to further emphasise ownership. Following this, the child was told that another child would come tomorrow, and that if he or she wanted to, he or she could give away some of the stickers to this other child. The experimenter emphasised that the child did not have to give away any stickers, and explained that the child’s decision would be completely anonymous. The child was told that if he or she wished to give away any stickers, then these could be placed into the white envelope before posting it into a box. The stickers that the child wished to keep should be placed in the brown envelope. It was further explained that the experimenter and any parents present would turn away and cover their eyes during the division. Three control questions were asked to ensure comprehension: “Where will you put the stickers that you want to keep?” “Which envelope belongs to the other child?” and “Do you have to give away any stickers?” Children who failed either of the control questions were excluded.

### Data coding and preliminary analyses

Children’s sharing behaviour was coded live and from videotape. The number of stickers shared was converted to a percentage for each child. Child age in months at the date of testing was calculated and included as a variable in all analyses. This was done to control for any potential variance associated with age within the sample. Two dependent measures were calculated from the sharing data. 1) The number of children deciding to share in each priming-condition (i.e. sharing nothing vs. sharing one or more stickers) was analysed using a logistic regression. 2) The percentage of stickers shared was analysed with an ANCOVA. Prime type, was entered as a between subjects factor and age in months was entered as a covariate. Preliminary analyses indicated no effects of gender or sibling status in any of our analyses so these will not be considered further. All summary statistics are presented in Table 1, and the raw data file is available as supporting information (S2 File). Time spent on the priming-task did not differ significantly across conditions (*F*(2, 87) = .27, *p* = .77, $\eta_{p}^{2}$ = .01, *M*_*self*_ = 239.83s, 95% CI _*self*_ = [236.28, 243.39]; *M*_*friendship*_ = 242.10, 95% CI _*friendship*_ = [237.14, 247.06]; *M*_*neutral*_ = 240.57, 95% CI _*neutral*_ = [235.51, 245.63]). Therefore, this factor will not be considered further.

**Table 1:** Summary statistics for sharing and helping data.

	**Study 1: Sharing**		
**Prime Type**	**Self**	**Neutral**	**Friendship**
N	30	30	30
Decision to share	22	28	28
% shared	19.44	35.56	34.44
[95% CI]	[14.00, 24.88]	[29.73, 41.39]	[26.97, 41.92]
	**Study 2: Helping**		
**Prime Type**	**Self**	**Neutral**	**Friendship**
N	30	30	30
Optimal helpers	6	18	17
Prompt score	2.07	1.63	1.57
[95% CI]	[1.61, 2.53]	[1.09, 2.17]	[1.03, 2.10]
Time helping (sec)	96.70	135.43	137.37
[95% CI]	[73.89, 119.51]	[112.43, 158.44]	[113.60, 161.14]
Stickers sorted	14.87	22.03	22.17
[95% CI]	[11.09, 18.64]	[18.17, 25.90]	[17.97, 26.36]

## Results

### Decision to share

Priming condition and age were not significant predictors of decision to share (X^2^ (3) = 6.655, *p* = .084), although there was a non-significant trend towards a reduction in willingness to share following self-priming compared to the neutral condition (β = 1.588, *p* = .060).

### Percentage of stickers

There was a significant effect of priming condition on percentage of stickers shared (*F*(2, 84) = 8.95, *p* < .001, $\eta_{p}^{2}$ = .18). Specifically, children shared a significantly smaller percentage of their stickers following self-priming (M _*self*_ = 19.44%), compared to neutral-priming (M _*neutral*_ = 35.56%, *t*(58) = 4.13, *p*< .001, *d* = 1.07, Bonferroni corrected), and friendship-priming (M _*friendship*_ = 34.44%, *t*(58) = -3.32, *p* = .006, *d* = 0.86, Bonferroni corrected). There was no significant difference in the percentage of stickers shared following neutral-priming and friendship-priming (t(58) = .24, p = .811, d = 0.06). A significant effect of age (*F*(1, 84) = 4.48, *p* = .037, $\eta_{p}^{2}$ = .051), indicated that older children shared a higher percentage of their stickers than younger children regardless of priming condition. There was no significant interaction between age and condition on sharing behaviour (*F*(2, 84) = 1.88, *p* = .160, $\eta_{p}^{2}$ = .043).

## Study 2: Helping

Overall, in study 1, completing a self-focused activity led to a reduction in sharing relative to the neutral condition; children shared a significantly smaller percentage of their stickers following self-priming. This may be because the natural development of sharing is to move from a position of self-interest to one that is more other-oriented. Thus, self-priming may have reverted participants back to a more egotistical position typically observed in younger children. There was no evidence that friendship-priming made children more generous in comparison to the neutral condition, although we did not have strong predictions in this regard. Helping, on the other hand, is evident much earlier in development, and as the nature of helping is arguably more other-focused, it may show greater sensitivity to manipulations that are designed to focus on affiliation with others. Moreover, some argue that helping is, in fact, a natural inclination [13] and so helping behaviour should be resilient to self-priming as there should be no self-focused bias related to helping. Thus, in Study 2, we sought to explore whether friendship-priming would boost children’s helping behaviour relative to self-priming and the neutral-priming task.

## Method

### Participants

Ninety 7-to 8-year olds (*M*_*age*_ *=* 95.92 months, *SD*_*age*_ *=* 6.97, range = 84–107 months; 43 males) participated in Study 2. This sample size was selected to match the sample size in Study 1. Six additional children were tested but excluded from analysis (a) because they were not in the right age group (n = 2), (b) due to interference from parents (n = 2), or (c) due to experimenter error (n = 2). All children were tested individually in quiet rooms at the Bristol Cognitive Development Centre or at the At-Bristol science centre. A majority of the children were of Western descent (96%) and came from middle-class, educated families. All ethical guidelines noted in Study 1 were also followed in Study 2.

### Procedure

Each session began by the child taking part in one of three priming conditions: self-priming, friendship-priming, or neutral-priming (control). The priming-tasks were identical to Study 1. Following the priming task, experimenter 1 (E1) stated: “Before you leave, I need your mum and/or dad to go to the room next door to fill in another form. You can just wait here with me. It will only take a few minutes”. A second experimenter (E2) led the parents out of the testing room. Next, E1 placed a bag with colourful animal stickers on the table whist explaining: “I use stickers for a different study with younger children, and while we wait I have to pick out all the frogs and elephants and put them into this box”. E1 then began to sort the stickers. If the child did not offer to help spontaneously, the experimenter waited for ten seconds before giving prompt 1: “There’s a lot of stickers for me to sort”. If the child did not join, the experimenter waited a further ten seconds before giving prompt 2: “This is going to take me a long time”. If the child still did not join, the experimenter waited a final ten seconds before giving prompt 3: “Could you help me a little”? A few moments after the direct request, E2 returned to the testing room, explaining that help was needed with the forms next door. Before leaving the testing room, E1 explained to the child: “We will be back soon. You don’t have to sort all of the stickers. Just do as many as you want and then you can leave the rest and pick out your prize from that bag over there” (pointing to a bag the opposite side of the room). After three minutes, E1 returned to the room, thanked the child for helping, and let him or her choose a prize if this had not already been done (see S3 File in supporting information for full experimental instructions).

### Data coding and preliminary analysis

The required number of prompts needed to elicit helping, time spent helping, and the number of stickers sorted was coded from videotape. Four dependent measures were calculated from the helping data. 1) Number of prompts needed before helping commenced ranged from 0–3 (0 = spontaneous helping; 1 = helped after the first prompt; 2 = helped after the second prompt; 3 = helped after direct request) and was analysed with an ordinal regression. 2) The number of optimal helpers (children who spend the full three minutes helping vs children who stop helping before instructed) was compared across conditions with a binary logistic regression. 3) The average time spent on the helping task was analysed with an ANCOVA. Prime type, was entered as a between subjects factor and age in months was entered as a covariate. 4) The number of stickers sorted was analysed with an ANCOVA, with prime type entered as the between subjects factor, and age in months entered as a covariate. All summary statistics are presented in Table 1, and the raw data file is available as supporting information (S4 File). Preliminary analyses revealed that time spent on the priming-task did not differ significantly across conditions (*F*(2, 87) = 1.29, *p* = .28, $\eta_{p}^{2}$ = .03, *M*_*self*_ = 236.23s, 95% CI _*self*_ = [233.67, 238.80]; *M*_*friendship*_ = 238.27, 95% CI _*friendship*_ = [236.41, 240.12]; *M*_*neutral*_ = 238.73, 95% CI _*neutral*_ = [236.05, 241.42]).

## Results

### Number of prompts & optimal helping

Priming condition and age were not significant predictors of the number of prompts needed before helping commenced (X^2^ (3) = 3.415, *p* = .332). However, the number of children who engaged in optimal helping was predicted by the priming-condition to which they were assigned (X^2^ (3) = 16.165, *p* = .001, *R*^*2*^ = .164). Specifically, fewer children helped for the full three minutes following self-priming (n = 6) compared to neutral-priming (n = 18, β = 1.992, *p* = .001). No significant difference was found between neutral-priming and friendship-priming (n = 17, β = .166, *p* = .756).

### Time on helping

Time spent on the helping task differed as a function of prime type (*F*(2, 84) = 4.52, *p* = .014, $\eta_{p}^{2}$ = .097). Children who completed self-priming spent significantly less time on the subsequent helping task (M _*self*_ = 96.70 seconds) compared to friendship-priming (M _*friendship*_ = 137.37 seconds), *t*(58) = 2.53, *p* = .042, *d* = 0.65, Bonferroni corrected). There was also a non-significant trend for children to spend less time on the helping task following self-priming compared to neutral-priming (M _*neutral*_ = 135.43 seconds, *t*(58) = 2.45, *p* = .054, *d* = 0.63, Bonferroni corrected). No significant difference in helping time was found between neutral-priming and friendship-priming (*t*(58) = .12, *p* = .91, *d* = 0.03, Bonferroni corrected).

### Stickers sorted

Finally, a significant effect was found of priming condition on number of stickers sorted (*F*(2, 84) = 4.49, *p* = .014, $\eta_{p}^{2}$ = .097). Children sorted significantly fewer stickers following self-priming (M _*self*_ = 14.87) compared to neutral-priming (M _*neutral*_ = 22.03, *t*(58) = -2.71, *p* = .027, *d* = -0.70) and friendship-priming (M _*friendship*_ = 22.17, *t*(58) = -2.65, *p* = .03, *d* = -0.68). No significant difference in stickers sorted was found between neutral-priming and friendship-priming (*t*(58) = .05, *p* = .96, *d* = 0.01).

Overall, the results from Study 2 replicated the negative effect of inducing self-focus on subsequent prosocial behaviour found in Study 1. Children were less likely to spend the full three minutes on the helping task and sorted fewer stickers following self-priming, compared to friendship-priming and the neutral-priming tasks.

## General discussion

The present study examined whether sharing and helping by 7-to 8-year olds could be influenced by a temporary activation of self vs. friendship concepts primed during semi-structured interviews. It was revealed that children in Study 1 shared a smaller percentage of their stickers following self-priming. In Study 2, children also spent less time on the subsequent helping task, and sorted fewer stickers following self-priming. Thus, self-priming reduced both sharing and helping behaviours compared to a neutral control condition. In the case of sharing, the results confirmed our prediction that self-focus would reduce the willingness of young children to donate stickers to another child. However, self-priming also reduced helping. Intriguingly, this finding could suggest that helping may be more self-focused than previously anticipated. Indeed, an uncharitable interpretation could be that helping behaviour has less to do with selflessly assisting another, and instead, is more strategic. Exploring this possibility goes beyond the scope of the current paper, but the interpretation is consistent with recent arguments by Dunfield and Kuhlmeier [50] and Wynn [51].

In contrast to self-priming, there was no evidence for an effect of friendship-priming on either sharing or helping. This is consistent with a growing literature on the dominance of self-oriented cognition such as the self-reference effect [52], but it still requires explanation. It is well established that pre-schoolers are more reluctant to share in comparison to other pro-social behaviours such as helping and cooperating (e.g., [18]). Thus, for sharing behaviour, the lack of a friendship-priming effect may not be too surprising because the current sharing task required children to give up resources without receiving anything in return, and their decision was kept strictly anonymous. As a result, the desire to keep more for oneself may have been too strong to be attenuated by the friendship-priming manipulation.

There are studies where friendship-priming has been shown to work. For example, it has been found that 3- to 4-year-old children primed to focus on their friends were more willing to trade toys with an experimenter [46]. One possible explanation for why friendship-priming worked for trading but not sharing is that these situations present the child with two very different predicaments. Only sharing presents a true cost. In the trading study, there was no real loss as the child was swapping for an identical object. Moreover, trading is a reciprocal activity whereas sharing is not necessarily so. In fact, the current Dictator Game involved sharing with an unknown child where there was no expectation to receive anything in return.

On the other hand, we predicted that helping would respond to friendship-priming because we considered it a more other-focused behaviour [53]. Moreover, it has previously been found that young children primed with stimuli that evoked feelings of affiliation were more likely to help a person in need compared to children primed towards individuality [48]. Consequently, we expected that focusing children on their relationship with a friend should evoke feelings of affiliation and lead to increased helping in a similar way. The absence of a friendship-priming effect for helping was thus surprising and worthy of further consideration.

Talking about friends could have evoked self-referential priming, as good friends can be considered an extension of one's self and may trigger a self-bias. However, if this was the case then there should have been a negative effect on prosocial behaviour relative to the control condition, which was not observed. A related point is that talking about a specific friend does not necessarily translate to prosociality directed towards others. We asked participants to talk about a specific friend because we reasoned that this would be a familiar concept for children of the current age group. Additionally, this enabled the experimenter to construct a picture based on the interview. Possibly, a more effective interdependence prime could have focused on others in general, or on mutual dependence and relatedness between individuals. Indeed, research indicates that people have universal and fundamental needs for relatedness and belonging that are essential to human development and growth [54], [55], [56]. In adults, it has been found that highlighting feelings of relatedness can lead to greater intentions to volunteer, and higher donations to charity [57], and that threatening the sense of belonging can decrease helping [58]. As children move in to a formal school setting, the experience of belonging, and the need to feel a sense of identification with others, become increasingly important (e.g., [59], [29], [60]). Thus, highlighting general feelings of connectedness, belonging and reliance on others may be a more powerful way of evoking prosocial actions in children. These issues may be worthy of future investigation, but we reiterate that our simple interdependence priming did work in our previous studies with younger children [46] and has also been shown to work in adults [37].

The striking effect from the current studies is that inducing self-focus through a simple priming task has a negative impact on prosocial behavior in young children. We consider this to be a significant contribution to the field of social development; especially given the paucity of developmental work using priming. However, the mechanism operating during self-priming is in need of further investigation. As previously discussed, it is possible that self-priming reverted participants back to a more egotistical position typically observed in younger children by boosting a self-interested bias. A potentially valuable avenue for future research would therefore be to explore whether children’s prosocial tendencies stabilize with age and become less vulnerable to self-priming. A related query is whether prosociality in younger children would be even more influenced by such priming effects. These are interesting questions, but it should be noted that the current study was not developmental, and it remains to be seen whether the same priming method would be as effective at different ages. We predict that priming will continue to influence children with increasing age as we note that adults are not immune to priming effects on prosocial behavior [41].

A further possibility is that the observed reduction in willingness to share following self-priming may be driven by an “endowment effect” [61]. The endowment effect can be described as a bias to over-value personal possessions. According to the “extended self” hypothesis, this overvaluation occurs because personal possessions are perceived as tangible manifestations of the self [62]. As people’s implicit self-evaluations are typically highly positive [63], this self-object association may lead to an enhanced perceived value of owned items [64], [65]. In support of this theory, it has been shown that the endowment effect can be strengthened in adults [66] and induced in young children [46] by focusing their attention on themselves. Children in Study 1 were endowed with stickers *before* entering the priming conditions so it is possible that the stickers in Study 1 were considered personal possessions. Therefore, the higher reluctance to give away stickers following self-priming could have been the result of an endowment effect. Future research could test this hypothesis by manipulating the time at which the children receive the stickers (i.e. before or after priming) and examine whether this attenuates the reduction in sharing.

While an endowment effect gives a neat mechanistic explanation for the reduction in sharing, it cannot explain why self-priming also led to a reduction in helping. Specifically, as helping involves relinquishing time and effort, rather than personal possessions, self-focus triggered biases in feelings of ownership do not predict changes in helping behaviour. Conversely, the interpretation that self-priming triggered a bias towards self-interest in both sharing and helping tasks provides a more parsimonious account.

It is also possible that self-priming task led to expectations of a positive outcome that were contradicted by the subsequent appeal to share or help. Indeed, if the self-focus interview was perceived as a positive experience that was “all about the child”, then the children may have been surprised by the request to help or share, which are activities that benefit others. Consequently, the resulting disappointment may have been reflected in their effort to help and willingness to share. Whilst this interpretation does not refute the main finding that self-focus significantly reduces sharing and helping, it could provide an alternative account of how self-priming operates.

Finally, we need to consider how the induction of self-focus can lead to different results on prosociality. For example, the presence of mirrors when performing prosocial acts has been shown to increase prosociality in both adults and children [67], [68]. Mirror reflections and talking about one’s self both trigger activation of the self-concept. Yet one context produces more selfless behaviour, whereas the other produces more selfish behaviour. We believe that reflections by mirrors can increase both private and public self-awareness and cause individuals to become highly self-conscious [69], [70], [71], [72]. This, in turn, will activate pro-social norms, which is also why both adults and children are more willing to make donations when they are being observed [73], [74], [75]. By contrast, in our studies, both sharing and helping was relatively unobserved as the experimenters turned their backs or left the room during the critical period so that children would not feel under pressure to conform to pro-social standards.

In conclusion, we have provided evidence that inducing self-focus in 7- to 8-year-olds can result in a reduced willingness to share and help. Children at this age are fully aware of, and adhere to prosocial norms [29], [30]. It is therefore noteworthy that they can be so easily influenced to become less prosocial by simply focusing on themselves. It may well be the case that the self-concept is easily triggered in childhood, whereas self-other construals may require stronger priming or may develop later as the child becomes more socially integrated with unfamiliar others.

## Supporting information

S1 File Priming scripts and experimental instructions for study 1: Sharing. (DOCX)

S2 File Raw data study 1: Sharing. (XLSX)

S3 File Experimental instructions for study 2: Helping. (PDF)

S4 File Raw data study 2: Helping. (XLSX)
