# Supplementary material for: Thinking of me: Self-focus reduces sharing and helping in seven- to eight-year-olds
Source: PLoS One. 2018 Jan 10;13(1):e0189752. doi: 10.1371/journal.pone.0189752 (PMC5761840; doi:10.1371/journal.pone.0189752)
Supplement: S1 File — (DOCX) [file pone.0189752.s001.docx]

**Supporting Information S1**

This file contains the experimental instructions given in study 1, as well as the full priming script used in both study 1 & 2.

**Study 1: Sharing**

**Experimental Instructions**

Sticker allocation

*Each testing session begins by presenting the child with a selection of 10 colourful, animal stickers.*

“Do you like stickers? I‘ve got 10 stickers here. And you get to choose 6 of them that you can take home with you. Which ones do you want to choose? OK great. These are yours, but for now, we will leave them up here on the table?” (*The experimenter moves the stickers out of reach on table*).

*Next, each child takes part in one of three priming conditions: self-priming, friendship-priming, or a neutral control condition. The priming-interviews were designed to take approximately 4 minutes to complete. A watch was used to ensure that this time limit was held as closely as possible (i.e., asking the child to elaborate if necessary/ appropriate).*

**Self-priming**

*The experimenter introduces the drawing game.*

“We’re going to play a game now. You can stay in your seat, but I’m going to move over here so I can’t see you”. (*The experimenter sits back to back with the child*).

“In my job, I am interested in how children describe themselves. So I want you to think about yourself, and I’m going to ask you lots of questions about you, what you look like, what you like to wear and the things you like to do and play with. And I’m going to draw a picture of you, based on your description of yourself. But I only have a few minutes to do so, so I’m going to start now”.

- What is the colour of your hair?
- Is your hair long or short?
- Is your hair curly or straight?
- Do you have a fringe?
- Do you have a favourite colour?
- Ok. Do you like to wear clothes in that colour?
- In this picture of you, what would you be wearing?
- What would your jumper look like?
- What would your trousers look like?
- How about your shoes? What colour are they?

“Ok, Now I want you to think about the things you like to do and play with”.

- Do you have a favourite toy?
- Have you had that for a long time?
- Do you have a hobby or are you part of any clubs?
- OK, how long have you been doing that for?
- So do you really like (e.g., football)?
- What else do you like to do when you’re not in school?
- Ok. What else do you like to play with?
- What about in school? What is your favourite subject?
- Why do you like that so much?
- Is there anything else you like to do or play with?

“That is all the time I have for my questions. Thank you very much for answering them”.

**Friendship-priming**

*The experimenter introduces the drawing game.*

“We’re going to play a game now. You can stay in your seat, but I’m going to move over here so I can’t see you (*The experimenter sits back to back with the child*).

In In my job, I am interested in how children describe their relationship with their best friend. So I want you to think about your best friend. Do you have a best friend? What’s the name of your best friend? OK. I’m going to ask you lots of questions about your friend and the things that the two of you like to do together, and I’m going to draw a picture of your friend and the things you do together based on your answers. But I only have a few minutes, so I’ll start now”.

- How long have you known ……..?
- How did you meet?
- What is the colour of his/her hair?
- Is his/her hair long or short?
- Is his/her hair curly or straight?
- Does his/her hair look anything like your hair?
- Does your friend have a favourite colour?
- Ok. Does he/she like to wear clothes in that colour?
- What would he/she wear in this picture?
- What would his/her jumper look like?
- What would his/her trousers look like?
- Do you remember what his/her shoes look like?

“Ok, now I want you to think about the things you and your friend like to play with, and the things you like to do together”.

- What is your favourite thing to do together?
- What else do the two of you like to play/do?
- Do you sometimes go to each other’s houses?
- What do you like to do when you visit each other?
- Do you go to the same school?
- What do the two of you like to do together in school?
- Can you think of anything else you two like to do together?
- What is it that makes ---- a good friend?

“That is all the time I have for my questions. Thank you very much for answering them”.

**Neutral-priming**

*Experimenter introduces the drawing game.*

“We’re going to play a game now. You can stay in your seat, but I’m going to move over here so I can’t see you (*The experimenter sits back to back with the child*).

In my job, I am interested in how children describe things. So I want you to think about a farm, and the animals that might live on a farm. I’m going to ask you lots of questions about the farm animals, and I’m going to draw a picture of the animals based on your answers. But I only have few minutes, so I’m going to start now.”

- Can you think of an animal that might live on a farm?
- Ok. What would the …. look like?
- Is it a big or a small ….?
- What colour is the …..?
- Can you think of another farm animal?
- Ok. What would the …. look like?
- And so forth.

(Questions were asked until a time limit of 4 minutes was reached).

“That is all the time I have for my questions. Thank you very much for answering them”.

Dictator Game

“I’ve got two envelopes here. One white and one brown. This is your envelope (brown). Everything you put in this envelope is yours and you get to take it home with you. I think we should write your name on it so we remember that you get to keep everything in it”.

“Now tomorrow, another child is coming here. This envelope is for that child (white). Everything that goes into this envelope is for that child and the child gets to take it home”.

“If you *want* to, you can give some of your stickers to the other child. You don’t *have to* give any away. But if you want to, you can. If you do want to give any away, then you can put them into the other child’s envelope and post it into this box”. (The experimenter shows the child a large, white post-box).

“If you look inside, you can see that the box is already full of other white envelopes. So no-one will know which one came from you” (demonstrates by opening the box). The stickers you want to keep you can put into *your* envelope”.

“Just to check that you understand the game, I’ll ask you some questions”:

- Do you have to share any stickers?
- Where will you put the stickers you want to keep?
- Which envelope belongs to the other child?

(*The order of the questions was counterbalanced*).

“OK, now I will turn around and close my eyes so I cannot see what you do with the stickers. It will be your secret. When you’re ready, put the other child’s envelope into the box, close your envelope, and let me know”.

After a few minutes, the experimenter asks if the child is finished. If the child confirmed this, the experimenter informs the child that the task is over and thanks him/her for helping. In addition to the remaining stickers, each child also receives a small prize for taking part.
