# Supplementary material for: Thinking of me: Self-focus reduces sharing and helping in seven- to eight-year-olds
Source: PLoS One. 2018 Jan 10;13(1):e0189752. doi: 10.1371/journal.pone.0189752 (PMC5761840; doi:10.1371/journal.pone.0189752)
Supplement: S3 File — (PDF) [file pone.0189752.s003.pdf]

Supporting Information S3

This file contains the experimental instructions given in study 2.

Study 2: Helping

Experimental Instructions

Each session begins by the child taking part in one of three priming conditions: self-priming, friendship-priming, or neutral-priming (control). The priming-tasks are identical to Study 1. Once the priming-task is completed, Experimenter 1 (E1) states:

"That's the end of my study. Thank you for answering my questions. Before you leave, I just need your mum/dad to go to the room next door to fill in one more form. You can just wait here with me. It will only take a few minutes".

Experimenter 2 (E2) leads the parent(s) out of the room. Next, E1 brings out a bag filled with colourful animal stickers. The bag is placed on the table precisely in the middle between the child and E1.

"While we are waiting, I just need to prepare my next study for today. I also do a study where I use stickers. I have to pick out all the frogs and elephants from this bag and put them into this plastic box".

E1 begins to sort the stickers. If the child does not offer to help spontaneously, the experimenter waits for 10 seconds before giving prompt 1:

"There's a lot of stickers for me to sort".

If the child does not join, the experimenter waits a further 10 seconds before giving prompt 2:

"This is going to take me a long time".

If the child does not join, the experimenter waits a further 10 seconds before giving prompt 3:

"Could you help me a little"?

A few moments after the direct request, E2 returns to the room stating:

"We're having some problems with the form. Could you please come and help us?"

Before leaving the room, E1 explains:

"I will be right back. You don't have to sort all of the stickers. Just do many as you want. Then you can leave the rest and go to pick out your price from that bag over there" (pointing to a bag of prizes placed within sight on the opposite end of the room).

E1 and E2 leaves the room and waits for 3 minutes before returning. When the 3 minutes are up, the experimenters and parent(s) re-enter the room, thanks the child for helping, and lets the child choose his/her prize if this has not already been done.
